# Case report: Iatrogenic tattoos caused by skin marking pen in a postoperative patient

**DOI:** 10.3389/fmed.2024.1387773

**Published:** 2024-04-11

**Authors:** Hanxing Zhao, Xingru Wu, Yue Yu, Chang Cao

**Affiliations:** ^1^Department of Burn and Plastic Surgery, West China Hospital, Sichuan University, Chengdu, China; ^2^Department of Dermatology, West China Fourth Hospital, Sichuan University, Chengdu, China

**Keywords:** surgical complication, pigmentation and colour, dermatosurgery, tattoo, iatrogenic accident

## Abstract

In this report, a female patient suffering from pigment retention caused by a skin marking pen was elucidated. The patient underwent blepharoplasty 6 months ago and presented with blue-black linear marks at the upper eyelid incision 2 weeks after surgery. Under dermoscopy, scattered pigments were observed to accumulate in the epidermis of the upper eyelid. The patient was diagnosed with iatrogenic tattoo by a surgical marking pen. We chose surgical excision of the skin with the pigmentation. Previous studies have established that the risk of bacterial contamination, contact dermatitis, and allergies may increase with the surgical marking pens, while pigment retention has not yet been mentioned yet. Here, we present a case with a pigment retention in the incision. The selection of the surgical labelling methods and the management of the pigmentation were also addressed. According to our clinical findings, the risk of pigment retention by marking pens needs to be mentioned in the patient’s informed consent. Therefore, the practitioner should ensure that the ink is cleaned by the end of each invasive procedure.

## Introduction

In dermatosurgery, oil-based surgical marking pens are involved in preoperative marking to define the surgical area. Optimally, the marker should withstand disinfectants and surgery wash without leaving a permanent stain on the skin. Increasing persistence and visibility, however, also increases the risk of colorant residue. Previous complications related to marking pens were mainly bacterial contamination, contact dermatitis, and allergies. No relevant literature has yet been reported on pigment residue. In our clinical practice, however, we have confirmed the possibility of oil-based markers leading to an iatrogenic tattoo at the time of surgery or injection.

## Case presentation

**Figure 1:**
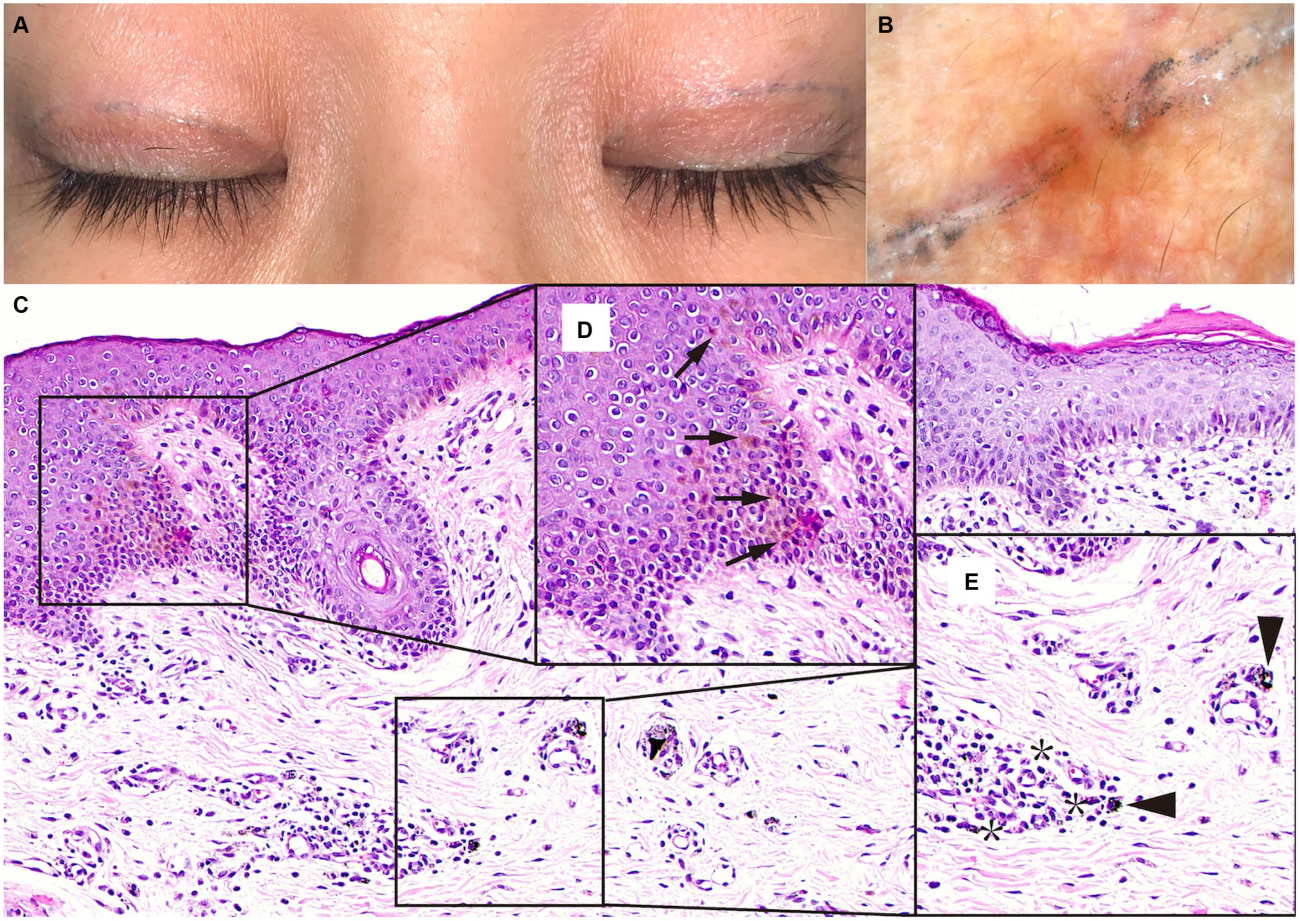
**(A)** Photograph taken 6 months after surgery showed an inhomogeneous blue-black pigment in the double eyelid incision. **(B)** The evaluation with a dermoscopy showed the exogenous blue-black pigments in the skin. **(C)** Histology of the upper eyelid skin tattooed with blue-black ink (H&E stain, magnification 20×). **(D)** Pigment particles are presented in the basal layer of the squamous epithelium (arrows), visible at 40× magnification. **(E)** Highly refractive brown pigment granules (triangle arrows) and chromophages (asterism) could be seen scattered in the superficial dermis and the perivascular area, visible at 40× magnification.

A 47-year-old female patient presented to the outpatient clinic with blue-black linear marks on the upper eyelid (Figure 1A). She had a blepharoplasty surgery 6 months prior. Two weeks after surgery, she found a blue-black linear mark on the bilateral upper eyelid. Conservative treatment was taken to monitor the changes in the marks. One month later, part of the pigmentation had faded, leaving some scattered pigments in linear arrangements around the incision.

On physical examination, the marks were approximately 7 mm from the supraciliary line hidden in the upper eyelid crease. The patient had no dermatitis over the pigment or keloid hyperplasia, hyperpigmentation, or depigmented changes in the surgical site involvement. On palpation, no foreign body was detected in the upper eyelid. Under dermoscopy, scattered pigments were observed to accumulate in the skin (Figure 1B).

The diagnosis of exogenous pigment residue was suspected, given her surgical history, physical examination, and dermatology examination. We speculated that the origin of the pigmentation was a surgical marker used in preoperative preparation. Our teams chose surgical removal of bilateral upper eyelid marks. The postoperative histological examination of the skin tissue confirmed that brown pigments accumulated in the squamous epithelium and the connective tissue (Figure 1C).

## Discussion

In preoperative preparation, marking pens are used to define the surgical field, design surgical incisions, mark landmarks, and others. Optimally, the markers should withstand disinfectants and surgical scrubbing without leaving a permanent stain on the skin. For visibility, the marker ink contains water-insoluble pigments that the body cannot degrade. Which means that increasing persistence and visibility also increases the risk of colorant residue.

Preoperative skin marking is an essential safety practice to prevent serious reportable events (SPE), as defined by the National Quality Forum (NQF) (1). However, unintended retention of a foreign object, including marking pen pigments in a patient after surgery or other invasive procedures, is also classified as an SRE. Previous studies have established that the risk of bacterial contamination, contact dermatitis, and allergies may increase with surgical marking pens (2–5). The terminology in the literature needs to be more consistent, and we refer to this complication as iatrogenic tattoo by marking pen.

The requirements for a skin marking pen are visibility and durability on the skin. Although oil-based pens are common, convenient, and inexpensive, the ink may contain carcinogenic substances such as crystal violet and xylene (6). Since conventional skin markers contain harmful substances, organic colorants have become increasingly used in skin marking. Studies on water-based ink have shown superior durability compared to the conventional oil-based pen (5). Moreover, Goto et al. (7) reported an organic-based marker using Inkbox^®^ (Inkbox, Toronto, Canada) with a duration of 16 days compared to 4 days for an oil-based pen. However, although the organic-based markers do not contain carcinogenic substances, some patients have experienced severe contact allergies or been provoked to granulomatous reactions (5, 8). The labeling approach differs with respect to skin types and the purpose of the labeling. For areas with hair growth, marking pens are more suitable than stickers. Similarly, marking pens are more suitable for marking the surgical field and incisions. Therefore, a balance should be struck between safety and effectiveness when choosing a proper marker.

Our patient’s presentation was colorant retention in the epidermis layer of the upper eyelids. In such patients with a sense of cosmetic issues, removing colorants needs to be addressed. The techniques listed in the published work include dermabrasion, cryotherapy, laser, and surgical excision (3). Dermabrasion and cryotherapy completely remove the epidermis to induce re-epithelialization, which is technique-dependent and time-consuming, with potential complications, including pigment changes, hypertrophic scarring, and infection. The introduction of newer therapies, such as lasers, allowed for precise determination of percent surface area and clinical recovery time and efficacy, providing more significant clinical outcomes. However, such procedures can hardly achieve removal effectiveness with a single treatment. Moreover, the safety of using lasers in the periorbital area is yet to be discussed. As aesthetics is the primary endpoint, surgical excision seems to be the preferable option for this patient.

In conclusion, according to this case report the marking pen may lead to iatrogenic tattoos. Physicians should inform patients of the risk of pigment residue before invasive procedures and avoid any pigment retention by the end of the procedure.

## Data availability statement

The original contributions presented in the study are included in the article/supplementary material, further inquiries can be directed to the corresponding author.

## Ethics statement

The studies involving human participant was reviewed and approved by the Medical Ethics Committee of West China Hospital of Sichuan University. All procedures performed in this study involving the human participant were in accordance with the 1964 Helsinki declaration. Written informed consent to participate in this study was provided by the participant. Written informed consent was obtained from the participant for the publication of any potentially identifiable images or data included in this article.

## Author contributions

HZ: Visualization, Writing – original draft, Writing – review & editing. XW: Conceptualization, Writing – original draft, Writing – review & editing. YY: Data curation, Writing – original draft, Writing – review & editing. CC: Supervision, Validation, Writing – original draft, Writing – review & editing.
